# Male Breast Cancer Prognostic Factors Versus Female Counterparts with Propensity Scores and Matched-Pair Analysis

**DOI:** 10.7759/cureus.355

**Published:** 2015-10-16

**Authors:** Edward Yu, Larry Stitt, Olga Vujovic, Kurian Joseph, Avi Assouline, Jawaid Younus, Francisco Perera, Patricia Tai

**Affiliations:** 1 Department of Oncology, Division of Radiation Oncology, Western University; 2 Clinical Research Unit, London Health Sciences Centre; 3 Radiation Oncology, London Regional Cancer Program, Western University; 4 Department of Oncology, Cross Cancer Center, University of Alberta; 5 Department of Radiation Oncology, Centre Clinique de la Porte de Saint-Cloud; 6 Medical Oncology, Western University; 7 Radiation Oncology, London Regional Cancer Program, Western University; 8 Department of Radiation Oncology, Allan Blair Cancer Center, University of Saskatchewan

**Keywords:** male, female, breast cancer, prognostic factors, propensity scores, matched-paired, survival

## Abstract

Objective: To assess the effect of prognostic factors and their impact on survival in male and female breast cancer.

Methods: Medical records for men and women diagnosed with breast cancer referred to the cancer center for treatment were reviewed. Patients with distant metastatic diseases were excluded. Data on prognostic factors including age, nodal status, resection margin, use of hormonal therapy, chemotherapy with and without hormone and radiation therapy (RT), survival, and recurrence were analyzed. Survival estimates were obtained using Kaplan-Meier methodology. The Cox regression interaction was used to compare male and female differences in prognostic factors. Male breast cancer (MBC) and female breast cancer (FBC) were matched according to propensity scores and survival compared using Cox regression.

Results: From 1963-2006, there were 75 MBC and 1,313 FBC totaling 1,388 breast cancers. The median age of the cohort was 53 (range: 23-90) years. Median follow-up was 90 (range: 0.4-339) months. Prognostic factors of patients were balanced among the groups after adjusting for propensity scores. A Cox model adjusting for propensity scores showed that overall survival (OS) (HR= 2.52 (1.65, 3.86), P<0.001) and distant disease recurrence-free survival (DDRFS) (HR= 2.39 (0.75, 3.04), P=0.003) were significantly different for MBC and FBC. Analyses that stratified by propensity score quintiles had similar findings: OS HR=2.41 (1.67, 3.47), P<0.001); DDRFS HR=2.89 (1.81, 4.60), P<0.001). When MBC and FBC were matched (1:3) by propensity scores, differences between MBC and FBC were again observed in OS (HR=1.94, 95%CI:1.18-3.19, P=0.009) and DDRFS (HR=2.79, 95%CI:1.36-5.75, P=0.005) with MBC at a higher risk of death and  disease recurrence compared to FBC .

Conclusion: This large series showed that MBC and FBC survivals are not similar, with MBC having a worse outcome. The finding of this study needs confirmation from a complete prospective database.

## Introduction

Male breast cancer (MBC) makes up fewer than 1% of all cancer cases in men and fewer than 1% of all breast cancer cases in the United States [1]. In 2014, 2,360 new cases were diagnosed in the United States and approximately 430 men died from this disease [2]. Because of its rarity, little is known about its etiology and there have been no randomized control trials of MBC. The management of MBC is primarily extrapolated from female breast cancer (FBC) trials and clinical data. Some investigators reported MBC has poor prognostic factors that may be responsible for survival outcome compared to FBC, others claimed equal prognosis for both sexes, and the controversy has not been completely resolved [3-10]. We have previously reported that prognostic factors influence survival differently in MBC and FBC [11]. The present study is to investigate the influence of prognostic factors when MBC and FBC are paired and matched in prognostic factors with propensity scores analysis.

## Materials and methods

Ethics approval was obtained through the Western University Health Sciences Research Ethics Board (approval #100929). Adult male and female patients with the diagnosis of invasive mammary carcinoma of the breast who were referred to the London Regional Cancer Program (LRCP) over the past 40 years were reviewed.

The patients were staged using the Seventh American Joint Committee on Cancer (AJCC) criteria for breast cancer [12]. Patients with Stage VI (M_1_) disease were excluded.

All patients received surgery consisting of either a lumpectomy and axillary dissection for breast preservation, a simple mastectomy and axillary dissection, or a modified radical mastectomy (MRM) for non-breast preservation management. Adjuvant radiation therapy was given in postoperative setting for high-risk patients with close/positive resection margins or tumor with positive nodes [13]. Radiation dose ranged from 40 Gy in 15 fractions to 50 Gy in 25 fractions to the breast or chest wall with or without supraclavicular axillary and internal mammary regions. A boost dose of 10 Gy in 5 fractions with electrons was generally given to patients with margin involvement. The radiation treatment energy was cobalt-60 or a 4-MV linear accelerator. Radiation treatment was given after the completion of chemotherapy.

Chemotherapy and tamoxifen were given in the adjuvant setting for high-risk patients with nodal involvement. The chemotherapy comprised of CMF (cyclophosphamide, methotrexate, and 5-fluorouracil), or CEF (cyclophosphamide, epirubicin, and 5-fluorouracil). Tamoxifen was also offered for estrogen receptor (ER) positive patients.

The primary endpoints for our review were overall survival (OS) and cancer-specific survival (CSS). The secondary endpoints were disease-free survival (DFS) and distant failure. Survival estimates were obtained using Kaplan-Meier methodology. Cox regression was used to compare male and female differences in prognostic factors. Male and female breast cancers were matched according to the propensity scores and survival compared using Cox regression.

To control for differences in the baseline characteristics, a propensity score was calculated for each male and female patient using a logistic regression model [14]. The model included patient cancer-related characteristics, such as age at diagnosis, tumor size, nodes removed, node positivity, resection margin status, hormonal treatment, chemotherapy treatment, and radiation treatment. We have incorporated changes in surgical technique by incorporating years of surgery (before and after 1987) in our propensity scores. Once the model was fitted, regression analyses were used to evaluate whether the baseline covariates were balanced across the study groups after adjusting for propensity scores. Potential time trends for cancer treatment, including the year of surgery in breast cancer management, was adjusted.

Cox regression analysis was used to compare the survival of female and male breast cancer, adjusting for propensity scores in three ways. First, the propensity scores as a continuous covariate in a Cox model comparing survival was included. Secondly, patients were classified into quintiles based on their propensity for gender and fitted stratified Cox model. Finally, patients were matched male to female by their propensity scores and compared survival among study groups [15-18].

## Results

From Jan 1963 to Dec 2006, a total of 1,388 breast cancer patient charts were reviewed. There were 75 MBC and 1,313 FBC. They were treated at a similar period of time; MBC were from 1979-2006 and FBC were from 1963-1992.

The median age of the cohort was 63 years (23-90 years): the median age for males was 65 years (range: 35-83 years) and for females was 60 years (range: 23-90 years).

The median follow-up time was 90 months (ranged from 0.39-339 months).

Patient characteristics included age, tumor size, tumor grade, nodal status, resection margins, hormonal therapy, chemotherapy with or without hormonal therapy, and radiation therapy, which were previously reported (Table 1) [11]. MBC patients were older in age (P=0.001) and their tumors were in the lower and intermediate grades (P=0.004). MBC tumor had a higher proportion ER positive (83% VS 57%), often treated with hormonal therapy only (P=0.001), and less often received chemotherapy-based treatment compared to FBC (P=0.001). After adjusting for propensity scores, all covariates were well-balanced among the groups and no clinically relevant differences between males and females were seen.

**Table 1:** Baseline Characteristics of Patients with Male Breast Cancer and Female Breast Cancer γ = Adjusted for patient propensity scores and with year of surgery. sd = standard derivation.

Characteristics		Male (n=75)	Female (n=1313)	P Value	
				Unadjusted	Adjusted^γ^
Age at Diagnosis					
	Mean (sd)	64.1 (11.6)	57.8 (12.2)	<0.001	0.304
	Missing	0	0		
Tumour Size (cm)				0.778	0.943
	≤2	36/68 (52.9%)	692/1254 (55.2%)		
	2-5	29/68 (42.7%)	489/1254 (39.0%)		
	>5	3/68 (4.4%)	73/1254 (5.8%)		
	Missing	7/75 (9.3%)	59/1313 (4.6%)		
Nodes Removed				0.149	0.522
	>10	37/72 (51.4%)	535/1252 (42.7%)		
	<10	35/72(49.6%)	717(1252(67.3%)		
	Missing	3 (4.0%)	61 (4.7%)		
Node positivity					
	Yes	38/75 (50.7%)	733/1313 (55.9%)	0.379	0.227
	No	37/75(49.3%)	580/1313 (44.1%)		
	Missing	0 (0.0%)	0 (0.0%)		
Margin Status				0.011	0.988
	Close/Positive (<2 mm)	11/60 (18.3%)	106/1222 (8.7%)		
	Negative (>2 mm)	49/60(81.7%)	1116/1222(91.3%)		
	Missing	15 (20.0%)	91(7.0%)		
Hormone Treatment				<0.001	0.501
	Yes	39/73 (53.4%)	220/1271 (17.3%)		
	No	34/73(46.6%)	1051/1271(82.7%)		
	Missing	2 (2.7%)	42 (3.3%)		
Chemotherapy				<0.001	0.315
	Yes	12/75 (16.0%)	597/1311 (45.5%)		
	No	63/75(84.0%)	714/1311(54.5%)		
	Missing	0 (0.0%)	2 (0.2%)		
Radiation Treatment				0.076	0.733
	Yes	46/75 (61.3%)	931/1312 (71.0%)		
	No	24/75(38.7%)	381/1312(29.0%)		
	Missing	0 (0.0%)	1 (0.2%)		
Year of Surgery					
	After 1987	62/75 (82.7%)	623/1313 (47.5%)	<0.001	0.155
	At and before 1987	13/75(17.3%)	690/1313(52.5%)		

The five-year and 10-year CSS and OS rates for node negative and positive patients are shown in Table 2.

**Table 2:** A. Male Breast Cancer (MBC) and Female Breast Cancer (FBC) Cancer-Specific Survival (CSS) with Time B. Male Breast Cancer (MBC) and Female Breast Cancer (FBC) Overall Survival (OS) with Time

A Male Breast Cancer (MBC) and Female Breast Cancer (FBC) Cancer Specific Survival (CSS) with Time					
CSS		5 years		10 years	
Negative nodes	MBC	94.5%		53.8%	
	FBC	91.8%		84.7%	
Positive nodes	MBC	79.4%		55.4%	
	FBC	72.6%		56.2%	
B Male Breast Cancer (MBC) and Female Breast Cancer (FBC) Overall Survival (OS) with Time					
OS			5 years		10 years
Negative nodes	MBC		68.7%		39.0%
	FBC		90.2%		85.3%
Positive nodes	MBC		74.6%		33.7%
	FBC		68.3%		49.2%

**Figure 1:**
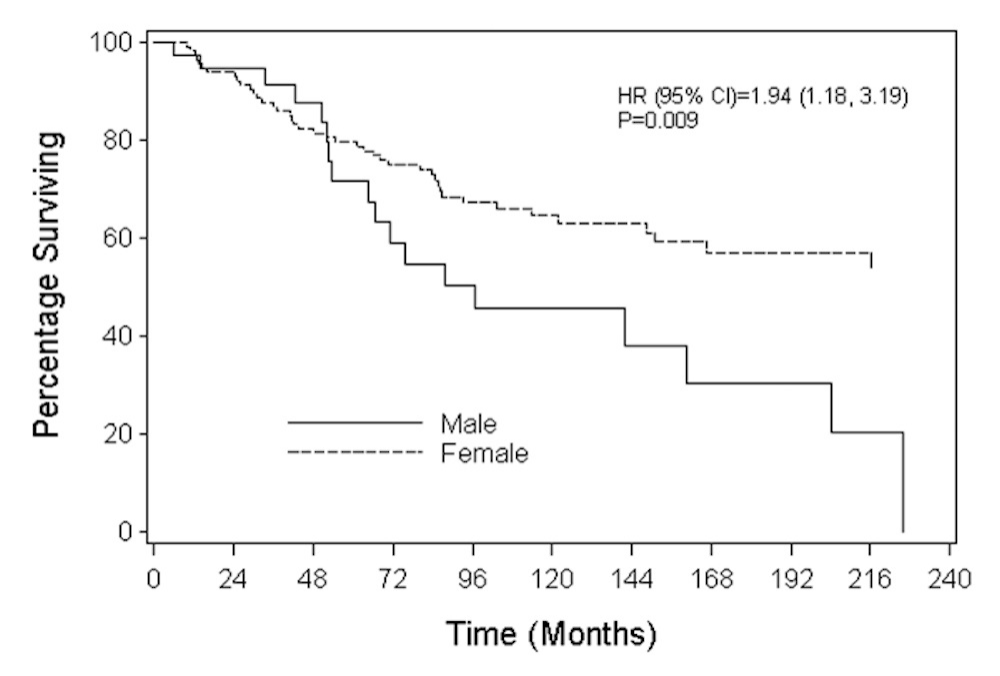
Overall Survival of Male Breast Cancer and Female Breast Cancer

**Figure 2:**
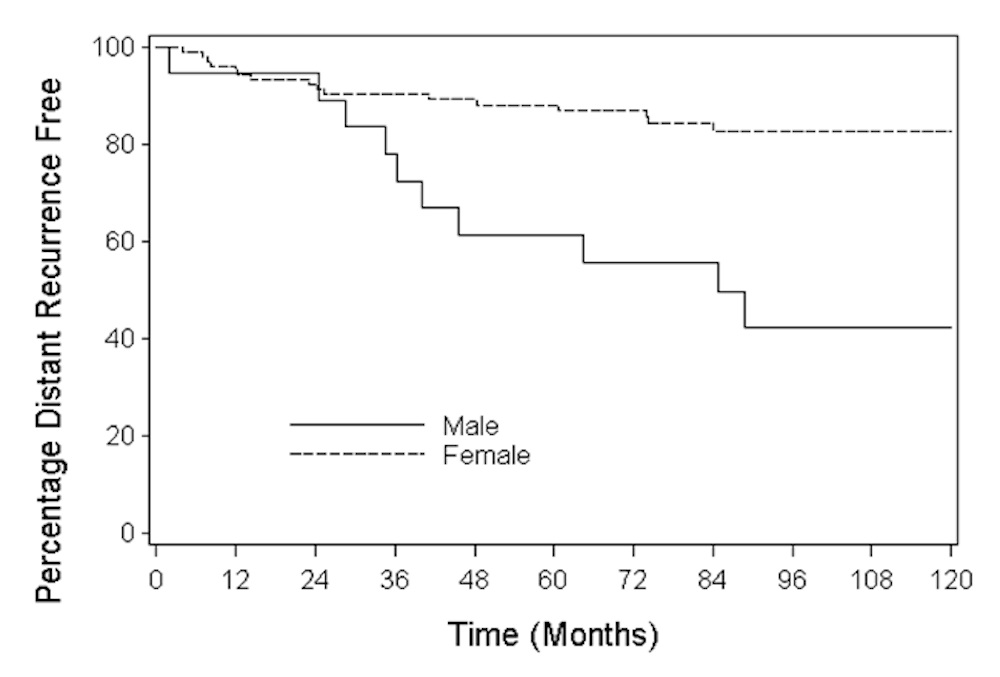
Distant Disease Recurrence-Free Survival of Male Breast Cancer and Female Breast Cancer HR (95% CI=2.79 (1.36, 5.75) P=0.005

Prognostic factors, including age, tumor size, nodes removed, resection margin status, hormonal therapy, chemotherapy, and radiation treatment were used for propensity scores analysis in male and female breast cancer (Table 3). A Cox model adjusting for propensity scores indicated that OS (HR= 2.52 (1.65, 3.86), P<0.001) and DDRFS (HR= 2.39 (0.75, 3.04), P=0.003) was significantly different for MBC and FBC. Analyses stratified by propensity score quintiles had similar findings (OS HR = 2.41 (1.67, 3.47), P<0.001); DDRFS HR = 2.89 (1.81, 4.60), P<0.001). Finally, when MBC and FBC were matched (1:3) by propensity scores, differences between MBC and FBC were again observed in OS (HR = 1.94, 95%CI:1.18-3.19, P=0.009) (Figure 1) and DDRFS (HR = 2.79, 95%CI:1.36-5.75, P=0.005) (Figure 2). For the subset of patients for whom estrogen receptors and tumor grades that were available, analyses were done and there was no difference between male and female breast cancer when adjusting for propensity scores. Both estrogen receptors and tumor grades did not contribute significantly to the models when adjustment was made. Analyses adjusting for potential time trends in the use of breast cancer treatment including surgery were employed, and the association between the MBC and FBC and survival differences remained unchanged.

**Table 3:** Propensity Score Analysis Compared of Overall Survival (OS) and Distant Disease Recurrence Free Survival (DDRFS) in Male Breast Cancer and Female Breast Cancer The HR represents the risk of death of a patient being male breast cancer (MBC) compared with a patient being female breast cancer (FBC)

	HR (95% CL), P value	HR (95% CL), P value
Entire cohort with adjusting for time trends and years of surgery	OS	DDRFS
Adjusting for propensity scores	2.52 (1.65, 3.86), P<0.001	2.39 (0.75, 3.04), P<0.003
Stratified by propensity score quintiles	2.41 (1.67, 3.47), P<0.001	2.89 (1.81, 4.60), P<0.001
Match-paired analysis(1:3) MBC:FBC Matched on propensity score with + 0.05 , 28 matched pair	1.94 (1.18, 3.19), P=0.009	2.79 (1.36, 5.75), P=0.005

## Discussion

Our data showed that MBC and FBC prognoses are not similar with the propensity scores analysis. MBC survivals are inferior compared to FBC. Different from FBC, but consistent with the literature, patients with MBC are older in age, tumor characteristics are more often with low to intermediate grades, and have a higher proportion of estrogen-positive receptors. In our center, they were often managed with tamoxifen rather than chemotherapy.

Although the prognostic differences between MBC and FBC are not entirely clear, potential under-treatment with systemic therapy and poor compliance to Tamoxifen in MBC have been reported [11, 19-20]. In our series, 50% lymph node involvement in MBC only 16% received chemotherapy whereas over 45% of FBC received chemotherapy. Research in progress is ongoing to improve patient care, including the development of specific geriatric screening tools to implement more aggressive systemic therapy to assist in breast cancer management who are older in age and at high risk for disease recurrence [21]. Further research is being directed at targeting estrogen receptor therapy in MBC, with preliminary data showing fulvestrant was an effective and safe treatment in hormone receptor-positive pretreated metastatic MBC [22].

Several limitations and strengths in our study are worth noting. Limitations include single center retrospective data that lacks patient co-morbidity information, limited ability to match all MBC with FBC for the specified criteria, and the patient cohort was not treated at the exact same period of time. However, we used propensity score methods to balance the study groups for covariates, including tumor size, nodal involvement, resection margin status, and cancer treatments, that are important prognostic factors for breast cancer patients receiving adjuvant therapy management. Although the controversy still remains, as others do not report a difference in MBC and FBC survival, our study is the first to match patient characteristics with propensity scores analysis and report that MBC differs from FBC in DDRFS and OS [23-25].

The International Consortium Study, which includes the International Male Breast Cancer Program, joint production of European Organization for Research and Treatment  of Cancer, Translational Breast Cancer Research Consortium, the Breast Inter-Group, and the North American Breast Cancer Group, has collected data for a retrospective analysis of all male breast cancer diagnosed and treated in the last 20 years. There is data for over 1,000 patients with M_0_ (no distant metastatic) disease. Tumor blocks were collected for the analysis of tumor biological characteristics, pathological review of ER, progesterone receptors, and HER-2 and Ki 67 expressions. The results of this International Consortium Study will hopefully provide further insight on MBC natural history and future management [26].

## Conclusions

This large series showed that MBC and FBC survivals are not similar. MBC differs from FBC in OS and in DDRFS significantly when matched by the propensity scores of their prognostic factors. Male patients with breast cancer are twice as likely to die of cancer than their female counterparts. The finding of this study needs confirmation from a complete prospective database.

## Appendices

Presented in part at the 56th Annual American Society for Radiation Oncology meeting at SanFrancisco , CA, Sept 2014
